# Pilot Study Evaluating the Feasibility of Comparing Computer Game Play with Close Work During Occlusion in Children Aged 2–7 Years with Amblyopia

**DOI:** 10.22599/bioj.132

**Published:** 2019-07-04

**Authors:** Catherine Jukes, Anne Bjerre, Jacqueline Coupe, Josephine Gibson

**Affiliations:** 1 Blackpool Teaching Hospital NHS Foundation Trust, GB; 2 The University of Sheffield, GB; 3 The University of Central Lancashire, GB

**Keywords:** amblyopia, occlusion, computer games, feasibility

## Abstract

**Background/Aims:**

Computer games have been used to stimulate vision in amblyopia with varying degrees of success. The aim of this pilot study was to evaluate the feasibility of conducting a randomised controlled trial to test the effectiveness of computer game play compared to close work during occlusion treatment in children.

**Method:**

Children aged 2–7 years with amblyopia and no prior amblyopia treatment were invited to participate. Participants were randomised to a computer game group or close work group and asked to complete two hours occlusion per day, incorporating one hour of their allocated activity. LogMAR visual acuity (VA) was assessed before treatment commenced and after 7(±1) weeks. The same examiner, who was unaware of the allocated treatment, assessed the participant using the same VA test.

**Results:**

Eighteen participants (mean age of 4.2 ± 1.3 years) completed the study. After seven weeks the mean VA of the amblyopic eye in the computer game group improved by 0.147 ± 0.182 logMAR, and in the close work group improved by 0.181 ± 0.124 logMAR. The difference in VA improvement between the computer game and the close work groups was not statistically significant (F_(1,32)_ = 3.71; p = 0.06).

**Conclusion:**

No significant difference was found in visual outcomes between the two groups, but a larger sample size would be needed to draw conclusions regarding the amblyopic population. Evaluation of the study design suggests it would be feasible to conduct a randomised controlled trial comparing computer games and close work during occlusion to determine if a significant difference in visual outcome exists.

## Introduction

There is growing research evidence that playing computer games can enhance visual abilities (Green & Bavelier 2012; Green, Li & Bavelier 2010; Hussain et al. 2014; Li et al. 2011). Several advances in computer technology have been explored in an attempt to actively stimulate visual acuity (VA) improvement in amblyopia. Perceptual learning on a computer involves extensive practice on a specific task, which has been shown to result in improvements in visual performance. This improvement is specific to the learned task, but it sometimes extends to improvements in visual acuity (Deveau, Lovcik & Seitz 2014; Polat et al. 2004; Zhai et al. 2013). There is evidence that amblyopia is a binocular condition caused by interocular suppression of the weaker eye (Birch 2013; Li et al. 2011). Dichoptic training is a binocular approach designed to reduce interocular suppression by allowing both eyes to contribute while playing specific computer games (Hess et al. 2014). The clarity or brightness of the image corresponding to the non-amblyopic eye is reduced, and certain elements of the task may only be seen with the amblyopic eye. Improvements in visual acuity have been found using this technique (Birch et al. 2015; Hess et al. 2014; Kelly et al. 2016; Li et al. 2013; Li et al. 2014; Žiak et al. 2017). The suggested mechanism of dichoptic training is that it reduces interocular suppression, yet some found no significant correlation between decreased suppression and increased visual acuity (Bossi et al. 2017; Kelly et al. 2016; Vedamurthy et al. 2015). Recent randomised controlled trials have found no benefit of dichoptic training compared with part-time occlusion (Holmes et al. 2016; Manh et al. 2018), placebo computer game treatment (Gao et al. 2018a), or glasses alone (Holmes et al. 2019). When considering strabismic patients, it could be argued that maintaining suppression is advantageous to prevent intractable diplopia. Computer game play with the non-amblyopic eye occluded has been shown to improve visual acuity in adults (Li et al. 2011) and children (Tailor et al. 2015). Action games have been found to result in the greatest visual improvement (Bayliss, Vedamurthy & Bavelier 2012; Li et al. 2009). A search of the literature did not reveal any full-scale randomised controlled trials testing the effectiveness of computer game play during occlusion in children.

A meta-analysis of studies comparing perceptual learning, dichoptic training and computer games found no significant difference in visual outcomes (Tsirlin et al. 2015). There was no significant difference in visual acuity outcomes using monocular or binocular viewing techniques. They concluded that so long as the amblyopic eye was given the chance to work, either alone or binocularly, vision would improve.

Specific elements of computer games are associated with stimulation, such as having levels to achieve, feedback with stars/rewards/bonuses and progression through the game. This stimulus, response and feedback presented in a visually complex and engaging way has been suggested as the reason for visual improvement (Jeon, Maurer & Lewis 2012). Rewards trigger neuro-modulatory learning signals such as acetylcholine, norepinephrine and dopamine, which are thought to influence plasticity and learning (Koepp et al. 1998; Rokem & Silver 2010; Seitz et al. 2006). Playing computer games uniocularly with the amblyopic eye has several advantages. Generally, no specialised software or hardware is required. Minimal intervention or supervision is required. Having a varied selection of games may increase the motivation to play, increasing game engagement and compliance. This could be achieved by employing a range of amblyopia specific games to provide greater variety (Tailor et al. 2015). Some of the games designed specifically for amblyopia treatment may be less successful due to the child losing interest after repeatedly playing the same game (Hussain et al. 2014; Holmes et al. 2016; Holmes et al. 2019; Gao et al. 2018a).

The aim of this pilot study was to evaluate the design of a randomised controlled trial to indicate whether one hour of computer game play during a two-hour occlusion period for amblyopia could be more effective at improving visual acuity in children compared to close work (reading/drawing). Close work was specified in group two as the viewing distance is comparable to tablet computer use, thereby reducing any potential distance-related variables. Additional objectives were to assess: the numbers of potential participants, recruitment and retention, the effectiveness of the compliance diary, the standard deviation of the measured effect (to calculate the sample size needed to produce meaningful statistical analysis), the success of masking the allocated treatment from the testing orthoptist and the acceptability of treatment to parents and children.

## Method

**Figure 1:**
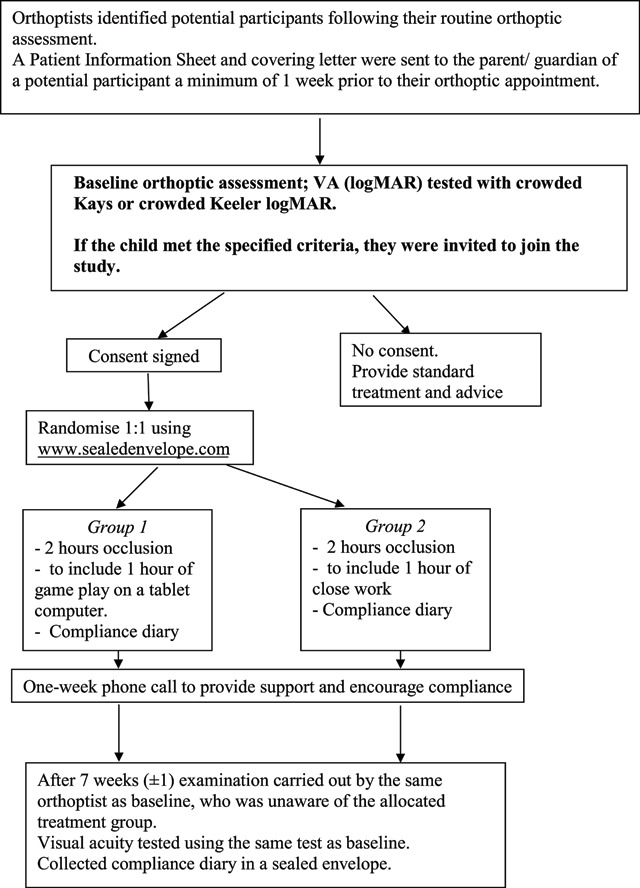
Flow chart.

The pilot study design was a prospective randomised controlled trial with two active parallel treatment arms. In the first treatment arm, participants were asked to play computer games on a tablet computer (iPad or Android tablet) for one hour of the two-hour occlusion period. In the second treatment arm, the participants were asked to incorporate one hour of close work (not involving a computer), into the two-hour occlusion period. Parents were asked to complete a compliance diary recording the duration and activity performed during occlusion. The orthoptist testing the participant at baseline and seven weeks was not aware of the treatment allocated. A flow chart of the study design is shown in Figure 1.

The primary outcome analysed was the change in best corrected VA of the amblyopic eye. Secondary outcomes were to assess the adherence to occlusion, the utility of the compliance diary, the acceptability of treatment, and to measure the standard deviation of visual acuity change to enable a sample size calculation.

The study was conducted at Blackpool Teaching Hospital NHS Foundation Trust between October 2016 and June 2017. Ethical approval was given by the Health Research Authority and London – Camberwell St Giles Research Ethics Council (REC – 16/LO/1496), the University of Central Lancashire Medicine and Health Ethics Committee, and Blackpool Teaching Hospital NHS Foundation Trust Research and Development department. This study adhered to the tenets of the Declaration of Helsinki.

### Participants

Any child with amblyopia (defined as two or more logMAR lines interocular acuity difference) was considered a potentially eligible participant. They were given or sent a patient information sheet by their orthoptist. If at their next routine assessment they met the eligibility criteria for the study, they were invited to participate in the trial.

### Inclusion criteria

- Children aged 2–7 years old.
- Presence of strabismic, anisometropic or mixed (both strabismic and anisometropic) amblyopia.
- Two or more lines difference in logMAR VA between each eye (using crowded Kays or crowded Keeler logMAR).
- Full ophthalmological assessment, correction of refractive error, and time for refractive adaptation (minimum of two months’ full-time glasses wear).
- Amblyopia stable after refractive adaptation (defined as less than 0.1 logMAR improvement in best corrected VA of the amblyopic eye at the first visit after completion of the refractive adaptation period).
- Best corrected VA of the amblyopic eye worse than 0.2 (logMAR).
- No previous occlusion treatment.
- Access to a suitable game platform (iPad or Android tablet computer), and the child and parent/guardian willing to be randomised to either treatment group.

### Exclusion criteria

- Insufficient cooperation with visual acuity testing or unable to perform the test reliably.
- Fundus and media abnormalities, nystagmus in primary position, or any other ocular or cerebral impairment.
- Orthoptist considers there is a risk of developing intractable diplopia (if the density of suppression tested using the Sbisa bar in any child aged five or over with strabismus was found to be less than 10).

### Consent and randomisation

Parents/guardians agreeing to their child’s participation provided written informed consent. In addition, age-appropriate information was provided for older children (aged 7), who participated only if they also assented.

To minimise selection bias, participants were randomised using an electronic randomisation service (www.sealedenvelope.com). Each participant had an equal probability of being assigned to either the computer game group or the close work treatment group.

### Intervention

1. Computer game group participants were given two hours occlusion per day to include one hour of computer game play using a tablet computer. Each participant was given a list of action computer games that were age appropriate and free to download (see supplementary file). They were encouraged to play one of these or choose their own game.
2. Close work group participants were given two hours occlusion per day to include one hour of close work such as reading, drawing, colouring, jigsaws or games. Participants were asked not to play computer games or use a computer of any kind during the two-hour occlusion period.

In the second hour of occlusion, participants were free to choose any activity, so long as it was not computer related. Each participant was issued a compliance diary to record the duration of occlusion and the activities carried out. The supervising adult was asked to complete this as accurately as possible.

One week after the onset of treatment, the orthoptist contacted the parent/guardian by telephone. The purpose of this was to provide any support needed and encourage compliance with treatment.

The same orthoptist tested the participant at baseline and seven weeks post randomisation (+/–1 week). The same visual acuity test was performed (either crowded Kays or crowded Keeler LogMAR). The compliance diary was returned to the research orthoptist in a sealed envelope. The orthoptist testing the participant was unaware of the randomised treatment allocated and was instructed not to enquire regarding treatment. The parents and children were asked not to discuss treatment allocation with the orthoptist examining them.

Clinical data from the baseline and seven-week assessment was collected from the participants’ hospital notes by the research orthoptist using case report forms. Data was inputted into a secure database at the hospital study site. Each randomised participant was allocated a unique participant number to enable confidentiality and anonymity of the participant.

The sample size for this pilot study was determined pragmatically rather than on the basis of a formal sample size calculation. This pilot study was carried out during a research internship and the sample size was limited by time constraints in which to complete the study.

### Statistical Analysis

Outcome data were analysed using GraphPad Prism 7.04. All data were analysed on an intention-to-treat basis. The mean, standard deviation and 95% confidence intervals of the change in VA of the amblyopic and non-amblyopic eye from the baseline to seven-week assessment were calculated. A paired two-tailed t-test was used to assess whether this change was significant (defined as 0.05). Repeated measures ANOVA was carried out to evaluate whether there was any significant difference in the mean VA change between the computer game group and the close work group.

Compliance with treatment was calculated by adding the total reported wear of occlusion and dividing this by the total allocated treatment time. If participants hadn’t completed or returned the compliance diary, this was estimated based on parental reports of daily wear. As it was not possible to calculate exact wear for each participant, a grading of 1–5 was given (1 being less than 10% of allocated wear, and 5 being over 75%). The mean estimated grading for each group was calculated to compare compliance.

Acceptability of treatment was graded by the parent at the seven-week visit as 1–5, with 1 being ‘easy’ and 5 being ‘impossible’. The mean score for each group was calculated and compared.

## Results

### Participants

**Figure 2:**
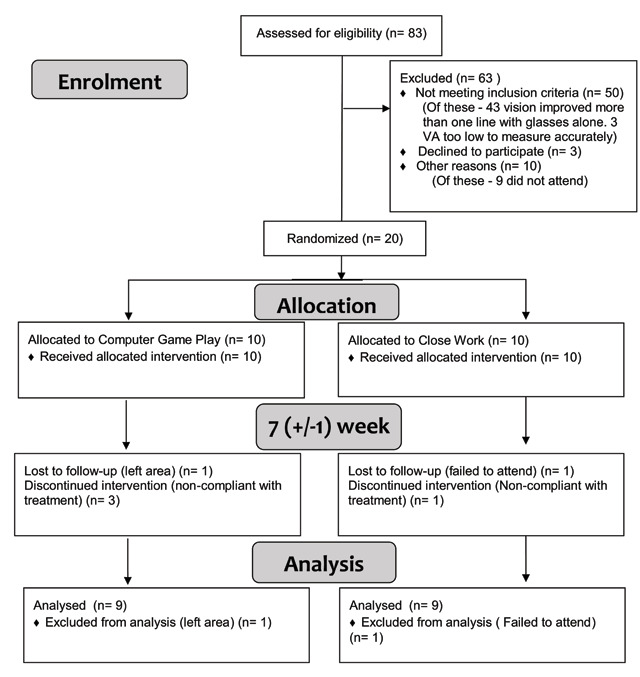
Consort diagram.

Eighty-three children were assessed for eligibility, of whom nine failed to attend their orthoptic appointment. Of the 74 attending, 50 did not meet the inclusion criteria. VA of the amblyopic eye had improved more than one line with glasses alone in 43 (58%) by a mean of 0.305 logMAR (varying from 0.100 to 0.750 logMAR improvement in an eight-week optical treatment period). Three children satisfied the criteria for enrolment but declined to participate. One child satisfied the enrolment criteria but didn’t satisfy study protocol (hadn’t had the specified time to consider the information prior to signing consent). Twenty participants were randomised between November 2016 and April 2017 (see Figure 2).

### Baseline Characteristics

All consenting participants were randomised, 10 to each treatment group. Two participants dropped out and did not complete the seven-week assessment (one moved out of the area, one failed to attend the seven-week appointment). Eighteen participants completed both the baseline and seven-week assessments, 7 male and 11 female. The mean age of participants was 4.2 ± 1.3 years, ranging from 2–7 years.

In the computer group there were only two male participants, compared to five in the close work group. There were five children with anisometropic amblyopia in the computer group, compared to one in the close work group (see Tables 1 and 2).

**Table 1:** Computer group participants amblyopic eye data.

Gender	Age (years)	Type amblyopia	VA (logMAR) baseline	VA (logMAR) 7 weeks	Change in VA (LogMAR)	Compliance (a)

female	7	anisometropic	0.350	0.225	0.125	poor
female	3	mixed	1.200	0.900	0.300	fair
female	6	anisometropic	0.300	0.350	–0.050	good
female	5	anisometropic	0.225	0.225	0.000	non-compliant
female	4	anisometropic	0.550	0.550	0.000	non-compliant
male	4	mixed	0.725	0.500	0.225	fair
female	3	mixed	1.000	.	.	dropped out
female	4	anisometropic	0.575	0.575	0.000	non-compliant
female	3	strabismic	0.750	0.250	0.500	excellent
male	5	strabismic	0.650	0.425	0.225	good

(a) Compliance: non-compliant <10%, poor 10–29%, fair 30–49%, good 50–74%, excellent 75–100%.

**Table 2:** Close work group participants amblyopic eye data.

Gender	Age (years)	Type amblyopia	VA (logMAR)	VA (logMAR) 7 weeks	Change in VA (LogMAR)	Compliance (a)

male	3	mixed	0.750	0.350	0.400	excellent
male	5	Strabismic	0.900	0.875	0.025	non-compliant
female	6	strabismic	0.600	0.400	0.200	excellent
male	2	strabismic	0.950	0.650	0.300	excellent
male	4	anisometropic	0.575	0.400	0.175	fair
female	5	strabismic	0.400	0.300	0.100	excellent
female	2	strabismic	0.325	0.175	0.150	fair
male	2	strabismic	1.000	0.750	0.250	good
female	7	anisometropic	0.400	.	.	dropped out
female	5	strabismic	0.875	0.850	0.025	poor

(a) Compliance: non-compliant <10%, poor 10–29%, fair 30–49%, good 50–74%, excellent 75–100%.

### Change in vision after treatment

The best corrected VA of the amblyopic eye at randomisation and 7(±1) weeks post randomisation for each group was analysed. Eighteen participants completed the trial, nine in each treatment group. The data of the two participants who did not attend the seven-week assessment were excluded from the analysis.

**Figure 3:**
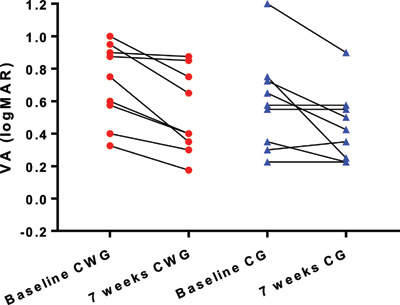
Visual acuity of amblyopic eye at baseline and seven weeks. Legend: CWG = close work group, CG = computer group.

**Figure 4:**
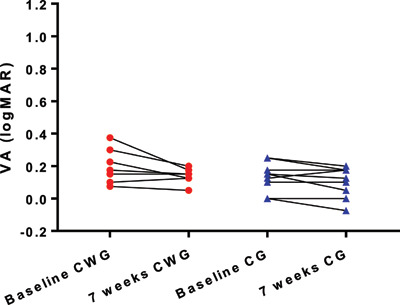
Visual acuity of non-amblyopic eye at baseline and seven weeks. Legend: CWG = close work group, CG = computer group.

The mean improvement in the amblyopic eye VA from baseline to seven weeks was 0.164 ± 0.152 logMAR (Figure 3). A paired t-test showed this was a significant improvement in visual acuity (p = 0.0003). There was also a statistically significant improvement in the non-amblyopic eye from baseline to seven weeks of 0.046 ± 0.060 logMAR (p = 0.005), although this did not reach clinical significance (defined as 0.05 logMAR) (Figure 4).

**Figure 5:**
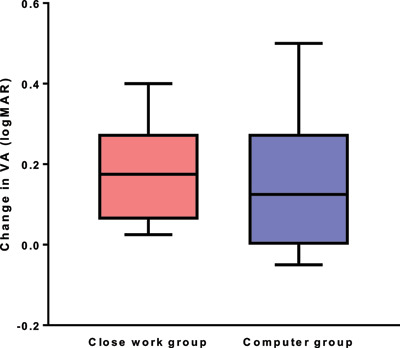
Change in visual acuity of amblyopic eye from baseline to 7(+/–1) weeks. Legend: The box plots represent the change in visual acuity in each group. The bottom and top of the box are the 25th and 75th percentiles. The solid lines extending up and down show the range of values. The line in the centre of the box shows the median value.

In the computer group, the mean amblyopic eye VA improved by 0.147 ± 0.182 logMAR (95% CI 0.008 to 0.287), ranging from 0.050 logMAR worse to 0.500 better. A paired t-test showed this was a significant change (p = 0.04). In the close work group, the mean amblyopic eye VA improved by 0.181 ± 0.124 logMAR (95% CI 0.276 to 0.085), ranging from 0.025 logMAR to 0.400 better. A paired t-test showed this to be a significant change (p = 0.02) (see Figure 5). Repeated measures ANOVA showed no significant difference in VA improvement between the computer game group and the close work group for both the amblyopic (F_(1,32)_ = 3.71; p = 0.06) and non-amblyopic eye (F_(1,32)_ = 2.69; p = 0.11).

The numbers of participants in this study were not sufficient to provide a detailed analysis of improvement with type or severity of amblyopia.

### Compliance with allocated treatment

**Figure 6:**
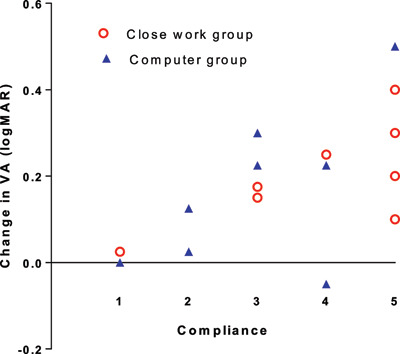
Change in visual acuity of the amblyopic eye compared to the level of compliance with prescribed treatment. Legend: The graph above shows the change in visual acuity of the amblyopic eye compared to reported compliance. 1 = non-compliant 0–10%, 2 = poor compliance 10–29%, 3 = fair 30–49%, 4 = good 50–74%, 5 = excellent 75–100%.

Of the 18 participants returning for the seven-week follow-up appointment, only 11 returned their diary (61%). Three reported they had completed the diary and would return it at a later date (yet failed to do so), and four reported they had not filled it in. For those who failed to return the diary, details of daily wear of occlusion were reported verbally to provide an indication of compliance. From the indicated wear, compliance was rated as 1 to 5, with 1 being less than 10% of allocated wear time, and 5 being over 75%. The mean compliance score was slightly better in the close work group (3.67 ± 1.50) than in the computer game group (2.67 ± 1.50). There was a positive correlation between reported compliance and improvement in vision (r = 0.69, p = 0.002) (Figure 6).

### Acceptability of treatment

Acceptability of treatment was rated by the parent on a scale of 1–5, with 1 being ‘easy’ and 5 being ‘impossible’. The mean score was similar in both groups (computer game group 2.56 ± 1.33, close work group 2.67 ± 0.87). Those finding the treatment ‘very difficult’ (4 on the scale) were all in the non-compliant or poor compliance categories. No parent rated the treatment ‘impossible’ (5 on the scale).

### One-week telephone call

At one week post-randomisation, a telephone call was made to the parent/guardian of every participant. This was generally successful, with the ability to reiterate the treatment plan, answer questions, and provide encouragement. A few participants were having trouble with occlusive patches, so a material patch was mailed as an alternative. These issues could have adversely affected compliance with treatment, so the short five-minute conversation was useful and worthwhile.

### Success of masking orthoptist

At the seven-week assessment, the testing orthoptist was unaware of the allocated treatment in 17 of 18 cases (94.4%). One participant inadvertently volunteered his allocated activity.

### Retention rates

Of the 20 participants recruited, one participant moved away during the study, and one participant failed to attend their seven-week appointment. This resulted in a 90% retention rate.

### Power calculation for future randomised controlled trial

The standard deviation was calculated from the results as 0.15 (s). A minimum clinically relevant difference between means of 0.05 logMAR (δ) was used based on a large study (Holmes et al. 2016). The sample size was calculated using the formula; n = f\left( {\alpha ,\beta } \right)*{\textstyle{{2{s^2}} \over {{\delta ^2}}}}.

In order to have 80% power for hypothesis testing, a total sample size of 142 participants per group would be needed. A total sample size of 314 participants (157 per group), would need to be recruited to take into account a 10% potential loss to follow up.

### Adverse effects

There were no adverse effects reported during this study.

## Discussion

The findings of this pilot study demonstrate that it is feasible to carry out a randomised controlled trial to assess the effectiveness of computer game play compared to close work during occlusion in children. Eighty-seven percent of participants eligible consented to take part in the study, and the dropout rate was low (10%).

### Reported Compliance

There was variability in the reported compliance in both groups, with five participants reporting no or poor compliance. The diary was only returned in 61% of participants. If a diary were to be utilised in a larger study, further development and testing would be carried out to improve its acceptability prior to the onset of the study. The desire of the supervising adult to be seen to comply with instructions might influence the accuracy of reporting in the diary, and verbal reports are possibly even less reliable. An objective measurement of compliance with treatment would be preferable, such as an occlusion dose monitor (Stewart et al. 2004b). The compliance rate reported in this study is similar to other occlusion studies (Holmes et al. 2016; Wallace et al. 2013). Reasons given in this study for not adhering to the prescribed amount of occlusion were: the participant didn’t tolerate the patch, difficulty fitting treatment into their daily routine (especially for working parents), holidays, birth of a sibling and illness. The participants in the close work group had the option of carrying out occlusion whilst at school. This may account for the slightly better reported compliance in this group.

Of those who returned their diaries, the percentage of time spent on the allocated activity during the occlusion period was 63% in the computer game group and 39% in the close work group. Although this might suggest that tablet computer games were easier to carry out than close work during occlusion, the overall adherence to treatment was better in the close work group.

One participant recorded 53% compliance in their diary, but unfortunately patched the wrong eye. This participant was therefore categorised as non-compliant with the allocated treatment. The information given to all participants (and all patients undergoing occlusion treatment) was subsequently altered to include a picture of which eye to occlude, as well as written instructions.

In both groups, better reported compliance correlated with greater visual acuity improvement. Compliance with allocated treatment was slightly better in the close work group, which may account for the slightly higher mean visual acuity improvement in this group.

### Variability of games/activities performed

The games and activities varied depending on the child’s age, gender and preferences. A list of age-appropriate action games was provided, and the participants were encouraged to play these. Some participants accessed alternative games that didn’t incorporate any fast-moving interactive elements. This may result in possible differences in stimulation as action games are thought to improve vision more effectively (Green & Bavelier 2012). For a future larger trial, specific games would be recommended.

### Difference in baseline characteristics

There may be variability in the improvements in VA depending on the initial level of vision, age and type of amblyopia (Stewart et al. 2005). Compliance with treatment was not found to be influenced by these factors (Wallace et al. 2013). A wide range of VA were measured at the baseline assessments. Participants with 2 lines VA difference could only achieve relatively small improvements, even though in one participant a 0.15 logMAR improvement resulted in clinically ‘normal’ vision. Successful treatment had been achieved, yet the analysis did not reflect this. In a larger study it may be necessary to stratify the sample to take this into account. Additionally, the percentage change in interocular acuity difference from baseline to seven weeks could be calculated for each participant, although this would not provide a directly comparable outcome measure.

The amount of occlusion prescribed (two hours per day) could be considered to be inadequate in participants with severe amblyopia. Occlusion dose-response studies have advocated larger doses of occlusion proportional to the severity of amblyopia (Stewart et. al. 2004b). For the purpose of this RCT, it was necessary to allocate the same dose to each participant to allow direct comparisons of the resulting changes in VA. Once a participant had completed the trial the occlusion dosage was increased if the VA improvement was unsatisfactory (although those adhering to the prescribed dosage in this study generally showed good improvement in VA).

### Changes in study processes

Early in the study a potential participant met the criteria but hadn’t been identified prior to attendance. They were not recruited as they would have had to return 48 hours later to satisfy the study consent processes. The time allowed to consider the information prior to signing consent was subsequently reduced from 48 hours, to enable consent on the same day if preferred (this was approved by the ethics committee). One participant had a reduction in VA despite good compliance with the allocated treatment. She was subsequently re-tested by the optometrist, who found a substantial change in her refractive error. This amount of change is unusual in a short time period. In any future research, a pinhole visual acuity would be recommended where practical to reduce the chance of this occurring. A minimum refractive adaptation period of eight weeks was specified in the inclusion criteria. It has been suggested that improvements in VA due to refractive adaptation may continue for approximately 14 weeks (Stewart et al. 2004a). A minimum of 18 weeks for refractive adaptation would be recommended in a larger study, as is now typically accepted best practice (Cotter et al. 2012; Gao et al. 2018b). Recruitment would have been improved if external clinics had been included in the protocol. For this pilot study there was insufficient staff, time and resources to accommodate this process. Twenty participants were recruited over a six-month period from November 2016 to April 2017. To satisfy the necessary sample size required for a full RCT a multi-centre trial would be essential. This would increase the complexity of study processes such as data collection.

Only those without prior amblyopia treatment were recruited to this study. This limited the number of potential participants. Occlusion is usually most effective at the onset of treatment, with effectiveness reducing after prolonged treatment (Stewart et al. 2004b, Holmes et al. 2016). The improvement achieved at different stages of treatment would therefore not be comparable.

## Conclusions

The results showed a significant improvement in mean VA of the amblyopic eye after treatment in both groups. Although the results suggest no significant difference between the computer game group and the close work group, a larger sample size would be needed to determine if this is applicable to the amblyopic population. The sample size calculated for adequate analysis was 314 participants (157 per group), taking into account a 10% potential loss to follow up. The findings of this pilot study demonstrated that it is feasible to carry out a randomised controlled trial to assess the effectiveness of computer game play compared to close work during occlusion in children. The data collection was practical and feasible without adding significantly to workload of clinicians.
